# Smelting Remains a Public Health Risk Nearly a Century Later: A Case Study in Pueblo, Colorado, USA

**DOI:** 10.3390/ijerph15050932

**Published:** 2018-05-07

**Authors:** Moussa M. Diawara, Sofy Shrestha, Jim Carsella, Shanna Farmer

**Affiliations:** 1 Department of Biology, Colorado State University-Pueblo, Pueblo, CO 81001, USA; shresthasofy@yahoo.com; 2 Department of Chemistry, Colorado State University-Pueblo, Pueblo, CO 81001, USA; jim.carsella@csupueblo.edu; 3 Regional Access to Graduate Education, Colorado State University-Pueblo, Pueblo, CO 81001, USA; shannamfarmer@gmail.com

**Keywords:** smelting, topsoil contamination, children blood lead level, *ALAD* polymorphism, public health risk, U.S. EPA Superfund National Priority List

## Abstract

Pueblo, Colorado has a long history of smelting activities, and recent studies raised concerns about lead exposure. This study tested 240 children in Pueblo for blood lead levels (BLLs) and found a significant association between distance from old smelters and children BLLs. Around 7.5% of Pueblo children had BLLs above the Centers for Disease Control and Prevention reference level of 5 µg/dL for elevated BLL, and 18.3% had BLLs between 3.3–4.9 µg/dL. Out of the 36 children who lived near former smelters, 13.9% had BLLs above 5 µg/dL vs. 6.37% for children living away from old smelters. The proportion of Pueblo children with elevated BLL was nearly three times the 2007–2010 United States national average (7.5% vs. 2.6%), and this was higher in the immediate vicinity of old smelters (13.9% vs. 2.6%). Genetic polymorphisms for *ALAD-1* or *ALAD-2* alleles, which play a role in susceptibility to lead toxicity, were not associated with children BBLs. Around 38.5% of houses sampled near the smelters had topsoil lead levels higher than the Environmental Protection Agency’s benchmark of 400 mg/kg. Our study resulted in the addition of areas of Pueblo to the EPA Superfund National Priorities List in December 2014, and cleanup is currently underway to minimize the public health risks.

## 1. Introduction

The city of Pueblo, Colorado has had a long history of smelting activities [1,2]. From 1890 to 1921, five smelters operated in the central business area of Pueblo (an area known today as Bessemer). These included the Blend Zinc Smelter (also known as the United States Zinc Company), the Colorado Smelter (also known as the Boston & Colorado Smelter, or the Eilers Plant), the Massachusetts Smelter (also known as the Ill-Fated New England Smelter), the Philadelphia Smelter, and the Pueblo Smelting and Refining Company. In addition to the smelters, there was the Colorado Coal and Iron Company steel mill (also known as the Colorado Fuel and Iron Company steel mill or CF&I). The five smelters processed various types of ores to produce gold, lead, silver, copper and zinc. The CF&I steel mill, which was not a smelter, processed pig iron to produce steel. By 1918, three of the five smelters were either dismantled or had their activities gradually weakened in the state; this included the main smelter, the Colorado Smelter, which closed in 1908. The remaining two smelters were destroyed in 1921 by the Pueblo’s great flood of the Arkansas River, putting an end to all smelting activities in the city [1,2]. However, the CF&I steel mill remained in operation. CF&I was later renamed Rocky Mountain Steel Mill-RMSM and is now known as EVRAZ-Pueblo [3]. From the mid-1800s to early 1900s, residential houses were built around the CF&I to accommodate workers [4]. The land use pattern around the mill remains the same to date and the central business area is adjacent to some of Pueblo’s poorest neighborhoods based on US Census data [5,6], raising concerns for environmental justice. The ores used by the smelters contained high levels of metals; including iron, lead, copper, silver and zinc.

Since the 1980s, the CF&I plant has been processing scrap materials to make various steel products [1]. However, by-products of the smelting processes still exist in the Bessemer area of Pueblo, Colorado, especially, along the western side of South Santa Fe Avenue in the form of slag heaps. Between 1992 and 1995, the Hazardous Materials and Waste Management Division (HMWMD) of the Colorado Department of Public Health and Environment (CDPHE) conducted a small-scale site inspection in Pueblo to determine the hazardous elements in the waste and slag piles and at other sites in Pueblo with a history of smelting [1]. A total of 13 source samples were collected from suspected historical smelter sites and slag heaps. The soil lead concentrations during their study varied from 11.2 mg/kg to 318 mg/kg at the suspected sites and from 598 mg/kg to 7640 mg/kg at the waste slag plies.

Between 2004 and 2005, Diawara and colleagues conducted a systematic geochemical characterization of topsoil in Pueblo, Colorado for heavy metals, and generated prediction maps using a spatial analysis interpolation technique called kriging [4]. The maps modeled a pattern of distribution for arsenic, cadmium, lead and mercury in Pueblo, Colorado. The soil sampling showed that the actual range of lead levels in the soil varied between 18 mg/kg and 316 mg/kg, with the concentrations exceeding 300 mg/kg in many low-income residential communities. Though these soil lead levels were below the EPA’s benchmark of 400 mg/kg for cleanup, the authors raised concerns about potential environmental contamination and expressed the need for a more comprehensive approach to address public health risks in these communities.

Based on the report by Diawara and co-workers and concerns raised therein [4], the HMWMD of CDPHE conducted a study in June 2010; focused near the historical Colorado Smelter site, which has residential neighborhoods around it [7]. They sampled topsoil from four locations of waste piles and from 47 residential properties around the historical Colorado Smelter site. The results showed that all of the samples collected along the four waste piles had soil lead levels higher than the EPA’s benchmark of 400 mg/kg; these were 1280 mg/kg, 1470 mg/kg, 5420 mg/kg, and 8070 mg/kg. The residential soil concentrations varied from 36 mg/kg to 785 mg/kg. The residential soil lead levels decreased as the distance increased from the former Colorado Smelter site.

Several studies have shown the relationship between soil lead levels (ranging from 370 mg/kg to 2453 mg/kg) and blood lead levels (BLLs) in children in the U.S.; and that BLLs increased as children lived closer to the smelters, putting them a greater risk of lead poisoning [8,9,10,11,12,13,14,15]. Socio-economic demographics, age of children, ethnicity, children’s behavior, and age of housing have also been shown to contribute to high BLLs in children [9,16,17,18].

Lead exposure can have detrimental effects on neurodevelopment as well as behavioral and cognitive skills, especially in children, because their nervous system is still in the developmental stage [18,19,20,21,22,23,24,25,26,27]. Because of these adverse effects, BLL in children has been constantly monitored in the U.S. [21,22,26,27], and the reference level used to determine lead poisoning in children has been accordingly re-examined over time. In 1971, the US Surgeon General set 40 µg/dL as the reference point for an elevated BLL that could be associated with adverse effects in children [28]. In 1975, the Centers for Disease Control and Prevention (CDC) lowered this intervention level to 30 µg/dL. Following the examination of new evidence on the effects of lead toxicity, the reportable BLL was further lowered to 25 µg/dL in 1985, and then to 10 µg/dL in 1991 [28]. In 2012, the CDC recommended a reference level of 5 µg/dL to identify children with elevated reportable BLL [27,29].

Studies of lead toxicity to humans showed that the main binding ligand for lead in red blood cells is δ-aminolevulinic acid dehydratase (*ALAD*) [30,31,32,33]. Expression of *ALAD*, coded by the *ALAD* gene, is known to have been associated with lead toxicity. That is, the *ALAD* polymorphisms might make some children more vulnerable to lead toxicity than others [34,35]. Hence, *ALAD* polymorphism may play an important role in children’s susceptibility to lead toxicity. Some studies have shown that individuals with the *ALAD-2* allele have higher BLLs than individuals with the *ALAD-1* allele [35,36,37,38]. Other studies found no statistically significant association between *ALAD* polymorphism and BLLs [39,40,41]. Considering the role of the A*LAD* enzyme in lead toxicity and the conflicting findings whether *ALAD* polymorphism might make certain individuals more sensitive to lead toxicity than others [34,35], further research on the *ALAD* gene polymorphism may help understand a child predisposition to lead toxicity. Identification of the allele associated with high BLLs can help identify children who are at potentially greater risks because of their genetic profile.

Clearly, elevated topsoil lead levels have been linked to high BLL in children in the U.S. [8,9,11,12,13,14,15]. Previous research has shown elevated topsoil lead levels in some areas of Pueblo, Colorado [1,4]; and in some areas, the levels were recently found to be much higher than the EPA’s 400 mg/kg benchmark [7]. This raised concerns for the community, especially regarding lead exposure in children. The study by Diawara’s group suggested that low-income families live closer to the sites with the highest topsoil lead levels, resulting in potentially higher exposure to lead [4]. The authors suggested the need for a more comprehensive study, as well as further assessment of population exposure. In the current study, we follow-up on the aforementioned studies by conducting environmental sampling and BLL monitoring in Pueblo, Colorado children. In order to get a better understanding of potential genetic factors influencing blood lead toxicity in children, the current study also conducts the first analysis of the distribution of allele frequency of the *ALAD* gene in the Pueblo children population. To our knowledge, this is the first comprehensive lead study conducted in Pueblo, Colorado that includes BLL screening and examination for genetic predisposition in children.

## 2. Materials and Methods

### 2.1. Classification of Zones

**Figure 1:**
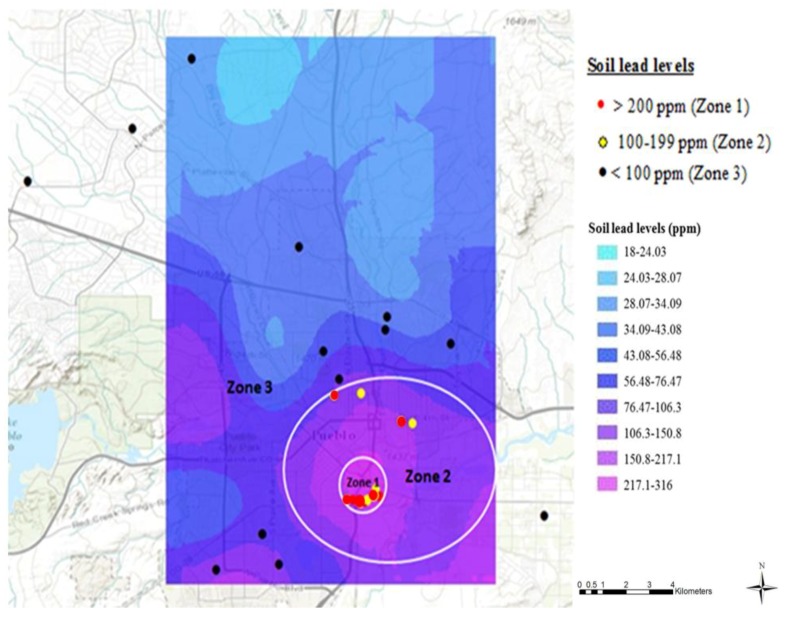
Categorization of Pueblo, Colorado based on topsoil sampling conducted during the current study in 2013 and re-constructed prediction map from previously reported topsoil lead levels [4]. All the data points in the figure represent the 31 actual topsoil sampling sites during the current study in the summer of 2013; these data are not based on previous studies or prediction maps. The city was categorized in three zones, based on predicted topsoil lead levels: Zone 1 with soil lead levels >200 mg/kg, Zone 2 with soil lead levels between 100–199 mg/kg and Zone 3 with soil lead levels <100 mg/kg. The main smelter, the Colorado Smelter, was located in Zone 1 and the other smelters were located in Zone 2.

We previously conducted a geochemical characterization of topsoil in Pueblo, Colorado to examine the levels of lead and other heavy metals, and generated spatial prediction maps [4]. These findings were used for the sole purpose of categorizing Pueblo into three different regions based on the lead content of the topsoil: Zone 1, Zone 2 and Zone 3 (Figure 1), in order to examine the impact of proximity to old smelters on BLL in children. All the data points in the figure represent the actual topsoil sampling sites during the current study in the summer of 2013; these data were not based on previous studies. Zone 1 comprises the main smelting area (0.75 km radius), around the Colorado Smelter, with soil lead levels predicted to be greater than 200 mg/kg; Zone 2 is farther away (0.75–3 km radius outside Zone 1) and included the other four smelters, with soil lead levels between 100–199 mg/kg; and Zone 3, the farthest from the smelting activities (more than 3 km radius outside zones 1 and 2), includes soil lead levels less than 100 mg/kg. 

### 2.2. Participants

The research was carried out in accordance with the Declaration of Helsinki, and the study protocol was approved by the Institutional Review Board of Colorado State University-Pueblo (CSU-Pueblo). Children aged 2–11 from different parts of Pueblo, Colorado participated in our study, during various lead screening events conducted from June 2012 to April 2013. Recruitment efforts included word of mouth, newspaper releases and flyers distributed around town by the Pueblo Community Action for a Renewed Environment (Pueblo CAREs), the CSU-Pueblo Child Care Center, and by the CSU-Pueblo students and staff. The lead screening events were conducted at the Zaragosa Reception Hall community center, the Saint Mary Help of Christians Church, the Pueblo Mall, the Rocky Mountain Service Employment Redevelopment Pueblo Head Start facility, and at the CSU-Pueblo Student Health Services. The parents of participating children received a $20/family compensation. Every parent was asked to show identification and to fill out and sign an informed consent form for publication before testing BLL of their children; parents received a carbon-copy of the consent forms they signed. Every parent also filled out a questionnaire requesting demographic characteristics including the home address, age of children, number of years the children lived in the house, children’s place of birth, the year children’s homes were built, children hand-mouth activity, whether children’s parents were informed about possible contamination in the area, and children’s medical condition. These forms were all approved by the CSU-Pueblo Institutional Review Board. Of the 240 children tested, 36 were from Zone 1, 87 from Zone 2, and 117 from Zone 3. According to the 2010 US Census [6], the population of Pueblo, Colorado was around 110,000 people, with 6.9% under five years old. The proportion of the population under 10 or 11 years old is not provided. Considering that 60% of the children who participated in our study were five years or under, we surveyed around 2% of Pueblo children five or under, plus an unknown proportion between 6 and 11 years old.

### 2.3. Blood Lead Testing

A LeadCare II Analyzer and LeadCare II Test Strips Kits (Environmental Science Associates Biosciences, Chelmsford, MA, USA) were used for the determination of lead level in the children’s blood. The detection limit of the unit was 3.3 μg/dL. The LeadCare II Analyzer and LeadCare II Strips Kits were all stored at room temperature, and blood lead level testing was also performed at room temperature. Calibration for accuracy was performed for each new lot of test strips as instructed by the manufacturer.

The blood samples for the determination of the BLLs were collected by registered nurses from the Pueblo City/County Department for Public Health and the Environment (PCDPHE) and graduate students from the CSU-Pueblo Nursing Department, under the supervision of the registered nurses. Gloves were worn at all times. All the children had their hands washed with soap and water, and then dried with non-recycled paper towel before testing. Children were then monitored to make sure they did not touch any surface before testing. 

A clean pad was laid on the blood collection area and was disposed of after each collection. A reagent tube was properly labeled with the child’s name and date of birth. The ring finger (or the middle finger) was gently massaged to warm and increase the blood flow and then pricked for blood collection, using a Unistik 2 gauge lancet (Fisherbrand #2202927; Fisher Scientific, Pittsburgh, PA, USA). 

Immediately after pricking, a heparinized capillary tube was filled with 50 microliters of blood. The capillary tube was placed in the reagent vial and a plunger was inserted into the top of the capillary tube to empty the entire volume (50 microliters) into the reagent vial. The reagent vial was capped and inverted 10 times to ensure the sample was completely mixed. A dropper was used to dispense a small amount of the mixture on the “X” spot of the sensor, which was read by the analyzer. The blood lead level was displayed on the analyzer after 180 s. All data were immediately recorded. After each reading, the used sensor was removed from the analyzer and discarded in a biohazard container. The Lead Care II analyzer can detect between 3.3 µg/dL to 65 µg/dL. “Low” in the display window indicates that the blood lead level of the sample is lower than the detection limit of 3.3 µg/dL. We used the Lead Care II analyzer because it has been shown to have an accuracy comparable to that of Inductively Coupled Plasma Mass Spectrometry (ICP-MS) for detecting BLL in children’s whole blood sample [42].

For our study, BLLs were divided into three groups: Children with blood lead levels <3.3 µg/dL were designated as undetectable; children with blood lead levels 3.3–4.9 µg/dL were designated as detectable; and children with blood lead levels ≥5 µg/dL were designated as reportable blood lead levels, since 5 µg/dL is the CDC reference for elevated BLL to be reported to the PCDPHE [27,29]. Parents were immediately informed about the BLL of their children and provided educational materials about the risks associated with lead poisoning and preventive measures. Children with BLLs of 5 µg/dL or higher were immediately referred to the PCDPHE.

### 2.4. Soil Analysis

During the summer of 2013, new soil samples were collected from the homes of children tested for BLLs, for the purpose of the current study. Samples were collected from the top 5 cm of soil in the middle of the front yard and/or the backyard of 31 houses in Pueblo, Colorado. Some houses had concrete or paved front yards, and some houses did not have backyards; these areas were not included. The geographical coordinates of each sample collection site were recorded. The samples were sieved through a 2 mm stainless steel mesh to remove small rocks and large paint chips. The sieve was washed and dried after each sample to avoid cross-contamination. Chemical analysis was conducted in the CSU-Pueblo Department of Chemistry using US EPA methods. Lead content was determined by ICP-MS using US EPA Method 6020a after digestion of the samples by US EPA method 3052 [43]. Briefly, 0.200 g of sample was weighed into a 30-mL quartz vessel for chemical analysis. Samples were rinsed from the side walls of the quartz vessel with a minimum amount of DI water. Afterward, 4 mL of concentrated HNO_3_ and 1 mL of HCl were added and samples were allowed to subside. Hydrogen peroxide (35%) (1 mL) was then added. Quartz vessels were placed in Teflon vessels containing 10 mL DI water and 2 mL 35% H_2_O_2_ the vessels were placed into a Milestone Ethos EZ microwave and heated to 180 °C. Samples were held at 180 °C for 10 min and allowed to cool. The Teflon vessels were removed from the microwave and diluted to a final volume of 10 mL with DI water. The samples were stored in polypropylene test tube for ICP-MS analysis. Rigorous quality assurance/quality control protocols [44] were followed, including insertion of “blind” standard reference materials for determination of the accuracy of the methods, and analytical duplicates to allow estimation of the precision of the method. Due to the nature of the soil in the sampling areas, spiked sample recovery was used as a substitute for standard reference soils. The relative percent differences in duplicates were only 3.5% while the overall standard recovery of spiked samples was 99.3%. Three measurements of soil lead levels were taken from each sample, and the average was calculated and reported.

### 2.5. Genotyping for ALAD Polymorphism

Samples for DNA analysis were collected by using Omni swabs buccal swabs. DNA was isolated using QIAamp DNA mini kits (Qiagen #51304; Qiagen, Germantown, MD, USA) following manufacturer’s protocol. Further purification of DNA samples was done using the glass milk method (GeneClean II Kit QBiogene, Inc. #1001-100; QBiogenel, Carlsbad, CA, USA). The DNA was then amplified using the primers identified by Shen et al. [45]. These were *ALAD* sense 5′AGACAGACATTAGCTCAGTA3′ and antisense 5′GGCAAAGACCACGTCCATTC3′. PCR was done using EasyStart PCR Mix-in-a-Tube (Molecular BioProducts, Inc. #6022; Fisher Scientific, Pittsburgh, PA, USA). PCR cycling parameters were optimized for generation of the 916 bp amplification product. The optimal cycling parameters were 1 cycle at 96 °C for 5 min; 33 cycles at 96 °C for 1 min, 56 °C for 30 s, and 72 °C for 1 min; 1 cycle at 72 °C for 5 min. Amplifications were then stored at 4 °C until analysis by restriction enzyme digest. The amplification product was double-digested with restriction enzymes Msp I and Pst I. After the digest was analyzed by agarose gel electrophoresis, and scoring of alleles was done by assigning *ALAD-1* allele to those samples showing a 584 bp band and *ALAD-2* to those showing a 513 bp band. Heterozygote genotypes were assigned to those with both 584 bp and 513 bp restriction digest products. 

### 2.6. Statistical Analyses

A Chi-Square test [46] was performed in order to determine the association between the BLLs of children and lead in the topsoil. BLLs were divided into three categories: number of children with BLLs <3.3 µg/dL, 3.3–4.9 µg/dL and ≥5 µg/dL (Table 1). Similarly, soil lead levels obtained during the current study were divided into three categories: Zone 1 (>200 mg/kg), Zone 2 (100–199 mg/kg) and Zone 3 (<100 mg/kg). A 3 × 3 (row × column) contingency table was created. The use of this two-way table Chi-Square test in our study was appropriate because the sample size was sufficiently large and each cell had an expected value of at least 5 (±20%). In addition, the null hypothesis (no relationship between blood lead levels of children and lead levels in the soil) was of equal rates or risks in all categories. 

The questionnaire answers collected from the parents were also categorized for the Chi-Square test in an attempt to determine any potential associations between BLLs of children and the age of children, number of years children lived in the house, children’s place of birth, the year children’s homes were built, children hand-mouth activity, whether children’s parents were informed about the possible contamination in the area, and children’s medical condition. However, of all these questionnaire answers, only one category (place of birth; i.e., born in Pueblo versus elsewhere) met the criteria for a valid Chi-Square test. A Wilson-adjusted sample proportion was obtained to determine whether there was an association between presence of *ALAD1-1* or *ALAD 1-2* alleles and BLLs. In order to find the Wilson-adjusted sample proportion, the BLLs were categorized as <3.3 µg/dL (undetectable by the LeadCare II machine) and ≥3.3 µg/dL (detectable and reportable levels).

## 3. Results

### 3.1. Blood Lead Testing

Among the total number of 240 children tested in the Pueblo, Colorado area during the current study, 18 (7.5%) had BLLs above the CDC reportable 5 µg/dL threshold, 44 (18.3%) had BLLs of 3.3–4.9 µg/dL, and 178 (74.2%) had BLLs below our detectable limit of 3.3 µg/dL. Out of the 36 children who lived in areas closer to the old Colorado Smelter site (Zone 1), 5 (13.9%) had BLLs above the reportable 5 µg/dL, 6 (16.7%) had BLLs between 3.3–4.9 µg/dL, and 25 (69.4%) had BLLs below 3.3 µg/dL. Of the 204 children living away from this main old smelter (Zones 2 and 3), 13 (6.4%) had reportable BLLs, 38 (18.3%) had BLLs between 3.3–4.9 µg/dL, and 153 (75%) had BLLS below 3.3 µg/dL. Children BLLs during the study varied from undetectable (<3.3 µg/dL) to a maximum of 8.0 µg/dL.

**Figure 2:**
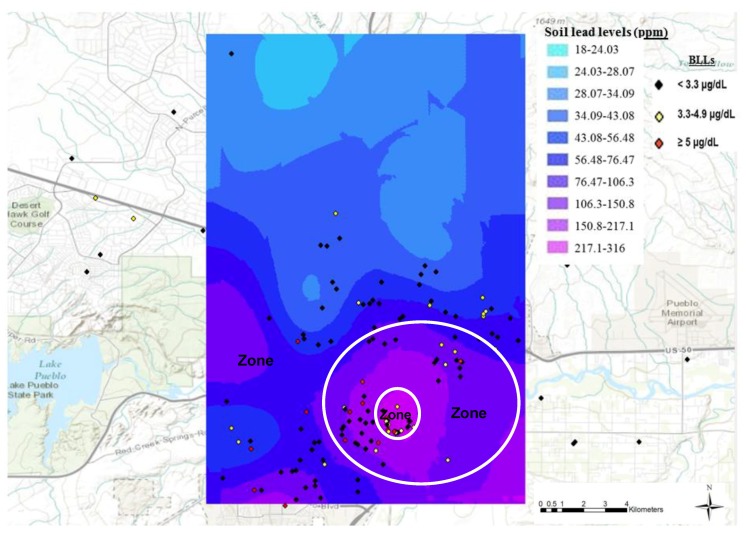
Blood lead levels and geographical coordinates of children superimposed on the spatial soil lead distribution prediction map. All the data points in the figure represent the actual geo-physical locations of the 240 children who were tested for blood lead level during the current study in the summer of 2013; these data are not based on previous studies or prediction maps. Each dot represents the site where participating children lived. The black, yellow and red colors represent <3.3 µg/dL, 3.3–4.9 µg/dL and ≥5 µg/dL BLLs, respectively. The main smelter, the Colorado Smelter, was located in Zone 1 and the other smelters were located in Zone 2.

When the children BLLs obtained during the current study and geographical coordinates of children physical addresses were superimposed on the spatial lead distribution prediction map (Figure 2), the children with BLLs ≥5 µg/dL BLLs were found primarily in areas with higher topsoil lead levels, around old smelter sites. The red diamonds show the children with BLLs ≥5 µg/dL, the yellow diamonds show children with BLLs between 3.3–4.9 µg/dL and the black diamonds show children with BLLs <3.3 µg/dL. As the map illustrates, the percentage of children with BLLs ≥5 µg/dL decreased as the distance from the old smelter sites increased. 

These observations are confirmed by the Chi-Square test which showed a statistically significant (*p* = 0.009; Table 1) association between the distance from the former smelting area and the categories of blood lead levels (undetectable, detectable, and reportable) of children in Pueblo, Colorado. Furthermore, Zone 1, which had predicted soil lead levels greater than 200 mg/kg, had 13.9% of children with reportable BLLs ≥5 µg/dL; this proportion is higher than in Zone 2 or Zone 3. Zone 2, where predicted topsoil lead levels varied between 100–199 mg/kg, had 9.1% of children with reportable BLLs, versus only 4.3% in Zone 3. The higher percentage of children with BLL between 3.3–4.9 µg/dL in Zone 2 may be explained, in part, by the presence of the other four old smelters in this zone.

Overall, the Chi-Square tests did not meet the criteria to perform a valid test for any factors on the questionnaire except children’s place of birth (i.e., born in Pueblo versus elsewhere). Of the 240 children who participated in the study, 196 were born in Pueblo. Of those children who had reportable BLLs (≥5 µg/dL), 94% (17 out of 18) were born in Pueblo. Similarly, of the children with BLLs between 3.3–4.9 µg/dL, 75% (33 out of 44) were born in Pueblo. Despite this trend, Chi-Square tests did not show a significant association between blood lead levels and children born in Pueblo versus elsewhere (*p* = 0.194).

### 3.2. Soil Analysis

When topsoil was sampled for heavy metals around 31 houses in the city of Pueblo during the current study, five of the 13 samples (38.5%) collected near the old Colorado Smelter (Zone 1) had topsoil lead levels higher than the EPA’s benchmark of 400 mg/kg, while all other samples were below this benchmark. The topsoil lead level distribution found during this study showed that all topsoil lead levels exceeding the EPA hazard standard for cleanup (>400 mg/kg) were found in the areas around the main old smelting site (Figure 1). The soil lead levels of the front yard and the backyard of houses often varied. Therefore, a mean value was calculated for each house. The highest mean soil lead level, 5120 mg/kg, was found at a house in Zone 1. Conversely, in the areas away from old smelters, the mean topsoil lead levels were all below 100 mg/kg. Soil lead levels of all samples collected during this study varied from 12 mg/kg to 10,011 mg/kg, with a median of 126 mg/kg and a mean of 366 mg/kg. The national US soil lead average is 19 mg/kg [4].

### 3.3. Genotyping for ALAD Polymorphism

A total of 231 children were tested for *ALAD* polymorphism. Of these, 13 (5.6%) had *ALAD1-2* polymorphisms, whereas 218 (94.4%) had *ALAD1-1* polymorphisms. None (0%) of the children who participated in the study had the *ALAD2-2* polymorphism. Thus, despite the relatively small sample size for genotyping, we were able to identify the *ALAD-2* allele in the heterozygous individuals of the population. Based on the Wilson-adjusted sample proportion, there were no significant differences between BLLs and presence of *ALAD-1* or *ALAD-2* alleles (Table 2).

## 4. Discussion

### 4.1. Blood Lead Levels in Children of Pueblo, Colorado vs. the U.S. National Average

This is the first comprehensive study that reports children’s blood lead levels along with the topsoil lead levels of these children’s residential homes in Pueblo, Colorado despite the long history of smelting activities in the region. The proportion of Pueblo children with reportable BLL in the zone near old smelters (13.9%) is much higher than the national average of 2.6% reported for US children by the CDC in 2007–2010 [27]. Overall, 7.5% (18 out of 240) of Pueblo children had BLLs above the CDC reportable threshold of 5 µg/dL, compared to the US national average of only 2.6%. Although the national average for US children with reportable BLL decreased from 8.6% in 1999–2002 to 4.1% in 2003–2006, and to 2.6% in 2007–2010, this trend is not seen in Pueblo. The proportion of Pueblo children with reportable BLL was more than two times higher when children lived near the main old smelter versus away (13.9% vs. 6.4%), further stressing the public health risks associated with these industrial activities. Our results are consistent with a recent report that 12.4% of 104 children tested in a Philadelphia community, with a history of lead-emitting industrial activities, had reportable BLLs or higher [47].

The CDC’s National Health and Nutrition Examination Surveys (NHANES) show a steady decline in BLLs in children in the U.S. [21,22,26,27]. The geometric mean BLL in U.S. children aged 1–5 years was 15.0 µg/dL during the 1976–1980 examination; this level dropped to 3.6 µg/dL during the 1988–1991 survey [21], and further dropped to 2.7 µg/dL by 1994 [22,26]. The geometric mean of U.S. children BLL had further dropped to 1.9 µg/dL by 2002 [26,27]; to 1.6 µg/dL by 2006; and to 1.3 µg/dL by 2010 [27]. The percentages of U.S. children aged 1–5 years with the current reference value of 5 µg/dL have also dramatically declined from 99.8% in 1976–1980 to 33.2% in 1988–1991, 20.9% in 1991–1994, 8.6% in 1999–2002, 4.1% in 2003–2006, and to 2.6% in 2007–2010 [21,26,27,48]. We were unable to determine the average BLL for all the 240 children tested in Pueblo during the current study because of the 3.3 µg/dL detectable limits of the LeadCare II Analyzer we used. However, it is alarming to note that the proportion of Pueblo children with reportable BLL (above the current reference value of 5 µg/dL) is currently nearly three times the US national average of 2007–2010 (7.5% vs. 2.6%), and this was even more dramatic for Pueblo children living in the immediate vicinity of old smelters (13.9% vs. 2.6%).

### 4.2. Association between Topsoil Lead Levels and Blood Lead Levels in Pueblo Children

The majority of the children who participated in this study were from the City of Pueblo (196 out of 240). Therefore, it is likely that the children who were exposed to lead were exposed via lead contamination in the Pueblo environment. Our results showed that the percentage of children with reportable BLL was the highest in Zone 1 (area near old smelters, with topsoil lead levels sometimes much higher than the EPA’s 400 mg/kg benchmark) when compared to Zone 2 and Zone 3 (13.9%, 9.1%, and 4.3%, respectively). This supports our hypothesis that children living closer to areas with high topsoil lead levels will have higher BLLs than children living in areas far away. Zone 2, where the other four smelters were located, had the highest percentage of children with BLL between 3.3–4.99 µg/dL. Overall, the BLLs in children decreased with the increase in distance from old smelters. This finding is consistent with reports by others [8,9,14,49].

Age can also play an important role in childhood exposure to lead, as certain behaviors result in the ingestion of lead from soil and paint chips. Exposure from topsoil as well as paint chips has been associated with BLL in children [50]. The percentage of children with BLLs ≥5 µg/dL in the current study was highest in children aged >5–6 years. This could be the result of greater outside play by this group or lead exposure from school or other public playgrounds. None of these areas were sampled during this study, and we did not ask parents about school frequentations. However, the Chi-Square test showed no association between soil lead levels and the age of children in this study (*p* = 0.253). Bradham and co-workers also found no significant correlation between age of children and BLLs [51].

The proximity of Zone 1 and Zone 2 to freeways, such as Interstate-25, might have played some role in higher lead levels in the residential sites of this study. Since Zone 1 and Zone 2 used to be Pueblo’s central business location, it is reasonable to state that heavy traffic flows in these areas might have resulted in heavier deposition from leaded gasoline. Even though the 1990 Clean Air Act and other EPA regulations banned leaded gasoline after 1995 [52], previous gasoline lead emissions could have already accumulated in the environment, especially in the soil. Others have suggested that lead exposure was directly associated with the degree of urbanization [49,53,54,55]. As mentioned in several of these studies, it is possible that in the current study, soil lead concentrations are further elevated next to homes because of the accumulation of lead aerosols on the sides of buildings, on trees, and on any vertical surfaces. This lead could be washed down into the soils by precipitation [54]. Since lead does not tend to leach into the subsoil, it can persist in the upper layer for many years [56,57]. So, some of the soil lead in this study could have come from the persistent presence of lead from a combination of smelting and the past use of leaded gasoline.

Studies in the past have shown that exterior lead paints on homes could also represent an important source of soil lead contamination [16,50,58]. The soil lead contamination in some residential areas in the current study could be due to the deterioration and flaking of exterior leaded paint, as well as renovation of housing containing leaded paint. Even though the use of leaded paint in residential buildings was banned in the United States in 1978 [59], many homes built before 1978 still have coats of lead-based paint, which continues to be an important source of soil contamination. In the current study, according to the questionnaire answered by parents, 39 of the 240 children who participated lived in houses built prior to 1950, 33 lived in homes built between 1950 and 1977, and 40 children lived in homes built in 1978 or later. Parents or guardians of more than half of the study participants (128/240) answered that they did not know the year their residence was built. Because the Chi-Square tests did not meet the criteria to perform a valid test for this factor, we were unable to draw any conclusions. However, the study found that the proportion of the 18 children with BLLs ≥5 µg/dL were higher in children living in houses built prior to 1950 (28%, 5/18), followed by houses built from 1950–1977 (17%, 3/18) and then houses built in 1978 or later (11%, 2/18). These findings are consistent with the NHANES’s 1999–2002, 2003–2006 and 2007–2010 estimated percentages of children 1–5 years of age with BLLs ≥5 µg/dL, which was found to be associated with the age of housing [27]. The association between age of housing and children’s BLLs could be due to exposure to soil contaminated from leaded paint used in the houses before the ban of lead in paint and past emissions of leaded petrol in older urban areas.

It is critical to note that the distribution pattern of the soil lead levels in this study show that all topsoil samples with lead levels higher than the EPA’s benchmark of 400 mg/kg are concentrated in the area with heavy smelting history (Zone I, Figure 1). This study revalidates the distribution of soil lead levels reported in 1995 by Buckingham [1], and in the 2006 study by Diawara and colleagues [4]. The topsoil lead levels decrease as distance increases from the areas with major past smelting activities. This result is consistent with other studies conducted around both active and inactive smelting sites [8,12,14]. Therefore, it is likely that past smelting activities are one of the main sources of soil lead accumulation in the area.

### 4.3. ALAD Polymorphisms and Blood Lead Levels

In our study, 94.4% (218/231) of children had *ALAD1-1* polymorphisms, 5.6% (13/231) had *ALAD1-2* polymorphisms, and none of the 231 children had *ALAD2-2* polymorphism. This is consistent with the fact that the general gene frequency of the *ALAD-1* and *ALAD-2* alleles is about 0.9 and 0.1, respectively [34]. Other studies have reported *ALAD-1* and *ALAD-2* allele frequency to be 0.88 and 0.12, respectively in a random normal Caucasian population [60]; 0.89 and 0.11, respectively in an Italian population [61]; and 0.98 and 0.02, respectively in a Taiwanese population [40]. The allele frequencies of the current study are close to those found in 236 children in China [62].

The lack of a significant association between BLLs and *ALAD* polymorphism during this study is consistent with previous research [39,40,41]. Hsieh and colleagues suggested that the lack of association between *ALAD-2* and high BLLs in their study could be because of their small size [40], which might be a possibility in this study as well. For a better understanding of the role of *ALAD* polymorphisms in lead toxicity, future studies should also examine association between *ALAD* polymorphism and other biomarkers, such as bone lead levels, dentin lead levels, and the determination of *ALAD* in both blood and urine. This might provide more parameters for the comparison, thereby determining the role of *ALAD* polymorphism in lead exposure in the children of Pueblo. A much bigger sample size might also increase chance of identifying children with the *ALAD-2* allele. Another limitation of the current study is the fact that we did not examine soil samples from schoolyards and public playgrounds as potential sources of childhood exposure to lead, nor did our survey questionnaire include school frequentations. Finally, the LeadCare II Analyzer we used could not detect BLLs lower than 3.3 µg/dL. Not only are lower levels of medical relevance, but a determination of BLLs lower than 3.3 µg/dL could have provided greater statistical power.

In summary, based on two recent reports [4,7], the EPA had a series of meetings with the Pueblo community leaders and local health departments to initiate discussions about listing areas in the southern part of the city on the National Priorities List (NPL) of the EPA Superfund [63]. The Superfund is a US Federal Program aimed at protecting public health and the environment through remedial efforts in abandoned hazardous waste sites. However, the Pueblo community leaders and health officials requested additional data (other than the EPA’s) to determine whether the former Colorado Smelter site and other nearby areas (referred altogether as Eilers) be placed on the NPL. The current study, which was already underway, served as an independent scientific evaluation. The findings reported herein were presented at various stakeholder meetings attended by community leaders in the Bessemmer area, and by the Pueblo City, the Pueblo County, the CDPHE, and the EPA to help make the decision to add the Eilers area of Pueblo, Colorado on the EPA’s National Priorities List. The results of our soil and children BLL analyses in areas of Pueblo clearly pointed to the need for remediation efforts. Consequently, the EPA added the former Colorado Smelter area to the National Priorities List of Superfund sites on 11 December 2014 [63]. Efforts are currently underway for cleanup of contaminated soils in the area. This study underlines the issues associated with the environmental pollution and degradation caused by lead-emitting industrial activities, and should serve as a warning for community leaders in both developed and developing nations in their decision to authorize these activities. As evidenced our findings, heavy metals such as lead do not biodegrade and remain a public health risk, even decades after cessation of the lead-emitting industrial activities.

## Figures and Tables

**Table 1:** Number of children in study Zones 1–3 (predicted high, moderate and low soil lead levels) found in the three categories of blood lead levels (undetectable, detectable, and reportable).

Zones of Lead Distribution in Topsoil	No. of Children with BLLs <3.3 µg/dL	No. of Children with BLLs 3.3–4.9 µg/dL	No. of Children with BLLs ≥5 µg/dL (Reportable)	Total Number of Children
Zone 1 (>200 mg/kg)	25 (69.4%)	6 (16. 7%)	5 (13.9%)	36
Zone 2 (100–199 mg/kg)	55 (63.2%)	24 (27.6%)	8 (9.1%)	87
Zone 3 (<100 mg/kg)	98 (83.7%)	14 (12.0%)	5 (4.3%)	117
Total number of children	178	44	18	240

**Table 2:** Wilson-adjusted sample proportion to determine association between *ALAD1-1*, *ALAD1-2* and blood lead levels. Though results are consistent with decreased blood lead levels among those with the *ALAD-2* allele, the numbers are too small to produce a statistically significant association.

BLL (µg/dL)	*ALAD1-1*	*ALAD1-2*	Wilson-Adjusted Sample Proportion
<3.3 µg/dL	157	12	95% confidence interval (−0.3305, 0.03350)
>3.3 µg/dL	61	1	
Total	218	13	
